# The Role of Monocarboxylate Transporters and Their Chaperone CD147 in Lactate Efflux Inhibition and the Anticancer Effects of *Terminalia chebula* in Neuroblastoma Cell Line N2-A

**DOI:** 10.9734/EJMP/2016/23992

**Published:** 2016-02-22

**Authors:** S. S. Messeha, N. O. Zarmouh, E. Taka, S. G. Gendy, G. R. Shokry, M. G. Kolta, K. F. A. Soliman

**Affiliations:** 1 College of Pharmacy and Pharmaceutical Sciences, Florida A & M University, Tallahassee, Florida 32307, USA.

**Keywords:** Plant ethanol extracts, monocarboxylate transporters, CD 147, lactate inhibitor, apoptosis, growth inhibition

## Abstract

**Aims:**

In the presence of oxygen, most of the synthesized pyruvate during glycolysis in the cancer cell of solid tumors is released away from the mitochondria to form lactate (Warburg Effect). To maintain cell homeostasis, lactate is transported across the cell membrane by monocarboxylate transporters (MCTs). The major aim of the current investigation is to identify novel compounds that inhibit lactate efflux that may lead to identifying effective targets for cancer treatment.

**Study Design:**

In this study, 900 ethanol plant extracts were screened for their lactate efflux inhibition using neuroblastoma (N2-A) cell line. Additionally, we investigated the mechanism of inhibition for the most potent plant extract regarding monocarboxylate transporters expression, and consequences effects on viability, growth, and apoptosis.

**Methodology:**

The potency of lactate efflux inhibition of ethanol plant extracts was evaluated in N2-A cells by measuring extracellular lactate levels. Caspase 3- activity and acridine orange/ethidium bromide staining were performed to assess the apoptotic effect. The antiproliferative effect was measured using WST assay. Western blotting was performed to quantify protein expression of MCTs and their chaperone CD147 in treated cells lysates.

**Results:**

*Terminalia chebula* plant extract was the most potent lactate efflux inhibitor in N2-A cells among the 900 - tested plant extracts. The results obtained show that extract of *Terminalia chebula* fruits (**TCE**) significantly (*P* = 0.05) reduced the expression of the MCT1, MCT3, MCT4 and the chaperone CD147. The plant extract was more potent (IC_50_ of 3.59 ± 0.26 μg/ml) than the MCT standard inhibitor phloretin (IC_50_ 76.54 ± 3.19 μg/ml). The extract also showed more potency and selective cytotoxicity in cancer cells than DI-TNC1 primary cell line (IC_50_ 7.37 ± 0.28 vs. 17.35 ± 0.19 μg/ml). Moreover, **TCE** Inhibited N2-A cell growth (IG_50_ = 5.20 ± 0.30 μg/ml) and induced apoptosis at the 7.5 μg/ml concentration.

**Conclusion:**

Out of the 900 plant extracts screened, *Terminalia chebula* ethanol extract was found to be the most potent lactate efflux inhibitor with the ability to inhibit chaperone CD147 expression and impact the function of monocarboxylate transporters. Furthermore, TCE was found to have growth inhibition and apoptotic effects. The results obtained indicate that *Terminalia chebula* constituent(s) may contain promising compounds that can be useful in the management of neuroblastoma cancer.

## 1. INTRODUCTION

Unlike normal cells, solid tumor relies on aerobic glycolysis as the primary source of energy, a phenomenon known as the Warburg Effect [1]. As the end-product of glycolysis, lactate is produced in an excessive amount [2] and considered an alternative source of fuel for the uncontrolled cell proliferation [3]. Lactate efflux to the cell microenvironment is critical to cell survival. The extracellular acidosis of the cancer cell was found to enhance cell invasiveness [4], metastasis [5], and chemotherapy resistance [6]. On the other hand, the continuous lactate production will cause intracellular acidosis. The acidic intracellular pH will eventually initiate apoptosis [7, 8] through different mechanisms such as promoting the permeability of mitochondria membrane [9], activating endonucleases that cause DNA fragmentation [10], or activating caspase-3 protease, the key indicator of apoptosis that deactivates essential metabolic proteins [11].

The mammalian cell has many transporters involved in the regulation of pH homeostasis [12]. However, monocarboxylate transporters (MCTs) are considered the most important pH cell regulators, especially within tumor cells with rapid metabolism and high glycolysis rate [13]. These MCTs (also known as solute carrier 16, SLC16 proteins) are a family of 14 transporters, and the first four members (MCT1-MCT4) documented as single-carboxylate molecules transporters across the biological membranes [14]. MCT1 is considered high-affinity lactate transporter involved in exogenous lactate uptake by the cancer cells [15] that facilitate lactate efflux according to pH gradient [16]. On the other hand, the low-affinity lactate transporters MCT4 release lactate [2]. Moreover, it was recently reported that MCT3 is involved in lactate efflux of some cells [17].

On the other hand, natural products have played a very important role as cancer chemotherapeutic agents [18]. Specifically, natural flavonoids were found as MCTs inhibitors [19]. MCTs are attractive targets for cancer therapy, especially in cancers of a hyper-glycolytic and acid-resistant phenotype [20]. Therefore, this study was designed to identify potent natural lactate efflux inhibitors among 900 plant extracts and to explore their mode of inhibition. Furthermore, the consequential effects of these extracts on cell viability, proliferation, and apoptosis were also examined.

## 2. METHODOLOGY

Screened plants and herbs were obtained from our “FAMU Herbal Resource Facility” where we have over 1100 stored medicinal plants. The facility is located within our research laboratory. The plants were originally obtained from several sources including Frontier Natural Products Coop (Norway, IA, USA), Monterey Bay Spice Company (Watsonville, CA, USA), Mountain Rose, Herbs (Eugene, OR, USA), Mayway Traditional Chinese Herbs (Oakland, CA, USA), Kalyx Natural Marketplace (Camden, NY, USA), Futureceuticals (Momence, IL, USA), Organic Fruit Vegetable Markets and Florida Food Products Inc. (Eustis, FL, USA). L-lactate assay kits were obtained from Eton Bioscience (San Diego, CA, USA), and water-soluble tetrazolium (WST) proliferation assay kits from GBiosciences (St. Louis, MO, USA). EnzChek® Caspase-3 Assay were purchased from Life Technologies Inc., (Grand Island, NY, USA). Resazurin (7-hydroxy-10-oxido-phenoxazin-10-ium-3-one), a-cyano-4-hydroxycinammic acid (CHC), phloretin and absolute ethanol were obtained from Sigma-Aldrich Co. (St. Louis, MO, USA). Other laboratory supplies were obtained from VWR International (Radnor, PA, USA), Atlanta Biological (Flowery Branch, GA, USA), and Santa Cruz Biotechnology, Inc. (Dallas, TX, U.S.A). Primary antibodies monocarboxylate transporter 1(MCT1), monocarboxylate transporter 3 (MCT3), monocarboxylate transporter 4 (MCT4), Basigin (CD147), and glyceraldehyde 3-phosphate dehydrogenase (GAPDH), secondary antibody and chemiluminescence reagent, were provided by Abcam (Cambridge, MA, USA). Pierce protein assay kit was purchased from Thermo Scientific (Rockford, IL, USA). Bio- Rad (Hercules, CA, USA) supplied running and transferring buffers, standard protein ladder, Laemmli sample buffer, and nitrocellulose. RIPA lysis buffer and mammalian protease arrest were obtained from G-Biosciences (St. Louis, MO, USA).

### 2.1 Plant Extraction

The screened plants were extracted with ethanol, the most common and safe organic solvents in pharmacological studies evaluating the activity of medicinal herbs [21]. Briefly, the selected plants were grounded, homogenized in 99.5% ethanol, and then placed in the dark on a shaker for 24 h at RT. Plant-ethanol mixture stored in air tight 15 ml glass containers at −20°C in the dark until the time of the study. Further, the identified plant extract for more investigation, Terminalia chebula fruits (TCE) was finely grounded and extensively extracted by soaking in 99.5% ethanol for seven consecutive days on a shaker in dark and at RT. The plant-ethanol mixtures were filtered and dried under vacuum, using a rotary evaporator below 40°C. The obtained crude ethanol extract of **TCE** was stored in the dark at −20°C for further studies.

### 2.2 Cell Culture

Mouse brain neuroblastoma cells (N2-A) and rat primary astrocytes (DI-TNC1) were purchased from American Type Culture Collection (ATCC, Manassas, VA). N2- A cell line used in the current investigation is a neuronal cell line known for its high lactate production compare to other cell lines. We, as well as others, have used this cell line and is considered an appropriate model to evaluate potential anti-cancer agents [22, 23]. We also used the N2-A cell line to investigate the “Warburg Effect” phenomenon [24], and cancer cells metabolism [25, 26]. On the other hand, the DI-TNC1 is an astrocyte immortal cell line with lower lactate efflux production compared to N2-A cells, an observation in our lab. The DI-TNC1 is very important in controlling brain energy metabolism [27, 28]. Cell culture Dulbecco's Modified Eagle Medium (DMEM), fetal bovine serum (FBS), penicillin/streptomycin, DPBS, and trypsin were all from Atlanta Biologicals (Atlanta, GA, USA). Cells were cultured in 75-cm TC flask at 37°C in humidified 5% CO_2_ incubator and were subcultured as needed with trypsin/EDTA. Growing media was supplemented with 10% FBS (v/v), 4 mM L-glutamine, and 1% penicillin /streptomycin.

### 2.3 High Throughput Screening for Lactate Efflux Inhibition

For screening plant extracts as lactate efflux inhibitors, N2-A cells (5×10^4^ /well) were seeded in 96-well plates and treated with 50 - 1000 μg/ml of plant ethanol extracts in a final volume 200 μl/well experimental media (phenol-free media supplemented with 1% each FBS/penicillin/streptomycin). Tested concentrations were determined based on previous preliminary studies. Control wells were treated only with ethanol at the highest used concentration (≤1.0%). After 4 h exposure period at 37°C and 5% CO_2_, 50 μl each of both experimental media and the lactate kit substrate mix were combined in another 96-well plate. The reaction was extended for 30 min at 37°C, CO_2_ - free incubator and stopped by 50 μl of 0.5 M acetic acid/well. The absorbance was measured at 490 nm using μQuant Monochromatic Microplate Spectrophotometer (BioTek, USA).

### 2.4 TCE Studies

#### 2.4.1 Lactate efflux assay

As lactate efflux inhibitor, the effect of **TCE** was compared to standard MCT inhibitors, phloretin, and α-cyano-4-hydroxycinammic acid (CHC). Based on previous preliminary studies in our lab, N2-A cells were exposed to gradual concentrations between 0 to 250 μg/ml. All experiments were performed at least two separate times with n=4, and the control cells were exposed to the used solvents at the highest tested concentration (≤1.0% of ethanol for plant extract or 0.1 % DMSO for standard inhibitors). Blank wells without cells were also included in the test.

#### 2.4.2 Cell viability assay

The redox dye resazurin was used for determining N2-A and DI-TNC1 cells viability after 24 h treatment with **TCE** at concentration range 0 – 250 μg/ml in experimental media. Control wells were treated only with ethanol at the highest used concentration (≤1.0%) and blank wells without cells were also involved in the test. In this assay, resazurin solution of 0.5 μg/ml in sterile phenol red free-phosphate-buffered saline (PBS) was used at concentration level 15% v/v. After an experimental period, the reduced resazurin was measured at 570 nm using μQuant Monochromatic Microplate Spectrophotometer (BioTek, USA). The percentage of N2-A cell survival compared to the control was calculated for IC_50_s determination.

#### 2.4.3 Western blotting

Neuroblastoma cells were plated in 6 wells plate at concentration 10^6^ cells/well and treated with low concentration of **TCE** (0-5 μg/ml) in the experimental media to keep cells alive and measure the changes in protein expression. Control wells were treated only with ethanol at the highest used concentration (0.1%) and blank wells without cells were also included in the test. After 4 h of incubation, cells were washed with PBS, pelleted and lysed for 30 minutes on ice with RIPA lysis buffer contains 1 X mammalian protease arrest. Samples were pulsed for few seconds with a probe sonicator and centrifuged at 10,000 ×g for 10 minutes at 4°C and the protein concentrations in cell lysates were determined using protein assay BCA. After that, the supernatant was diluted (1:1) with Laemmli sample buffer and boiled at 100°C for 3 minutes. Proteins from total cell lysates were loaded at consistent concentration 40 μg/ml and separated at 200 v constant voltages for 30-40 minutes using 10% SDS-PAGE gels and running buffer. Proteins were transferred to nitrocellulose membranes in the ice-cold transferring buffer for 90 minutes at 100 Voltage. Nitrocellulose membranes were incubated on a rocking shaker at room temperature for 1 hour with blocking buffer (5% non-fat dry milk in 1X PBST, pH 7.6) followed by 3x wash. All membranes were then incubated overnight with 10 ml of primary antibodies – diluted blocking buffer as following: MCT1 (1μg/ml); MCT3 (2.5 μg/ml); MCT4 (1:800); CD147 (1: 2,000) and GAPDH (1 μl/ml). After 3X wash with PBST, membranes were reincubated at RT for 3 hours with secondary antibody at dilution (1: 5,000). Finally, nitrocellulose membranes were washed with PBST and developed with chemiluminescence reagent. Images were captured using a Flour-S Max Multiimager (Bio-Rad Laboratories, Hercules, CA) and analyzed to obtain the band density with Quantity One Software (Bio-Rad Laboratories, Hercules, CA).

#### 2.4.4 Caspase 3 apoptosis study

Apoptosis study was conducted by assessing caspase -3- activity using EnzChek® Caspase-3 assay kit. Briefly, N2-A cells were seeded at an initial concentration of 0.5 × 10^6^ cell / well in 6 - well plates and treated with serial concentrations of **TCE** (0 - 30 μg/ml) in experimental media at a final volume of 3 ml/well. Tested concentrations were determined based on dose-response viability study. Control wells were treated only with ethanol at the highest used concentration (0.15 %) and blank wells without cells were also applied in the test. After 4 h incubation period, treated cells from each well were harvested, pelleted, washed in PBS. Cell pellets were resuspended in 50μL lysis buffer for 30 min on ice followed by centrifuge for 5 minutes at 4,100 ×g to pellet the debris. Lastly, 50 μl of each samples supernatant and the apoptosis kit substrate working solution were combined in another microplate well for 30 min at RT and the background fluorescence was determined by using 50 μL of the cell lysis buffer. Fluorescence intensity for each sample was measured (excitation/emission ~342/441 nm) using Synergy HTX Multi-Reader (BioTek, USA)

#### 2.4.5 Acridine orange / ethidium bromide apoptosis study

Acridine orange/ ethidium bromide staining assay was performed to detect apoptotic changes in N2-A cells. The applied conditions for the assay were similar to the caspase-3 apoptosis study. Monolayer treated cells were washed 3X with PBS and incubated with the stain for 30 min. The dyes were added to the cells in 1:1 ratio at a final concentration of 5mg/mL acridine orange and 3 mg/ml of ethidium bromide. The excess dye was removed, and cells washed 2X with PBS and imaged at 40X magnification using Nikon Eclipse Ti fluorescence microscope (Nikon Instruments Inc., Melville, NY, USA).

#### 2.4.6 Growth study and morphological changes

Cyto Scan™ water-soluble tetrazolium (WST 1) assay was used to measure growth rate in N2-A cells. Briefly, cells were plated at an initial density of 2 ×10^4^ cells / well in 96 well plate and treated with TCE at concentration range (0 - 60 μg/ml) in a final volume 200 μl / well phenol-free growing media. The tested concentrations were determined based on dose-response viability study. Control cells were exposed to 0.3% ethanol in culture media and corresponding blanks were performed as treatments without cells. After 48 h of incubation, cells were combined with WST 1/CEC assay reagent at 10% v/v for 30 min to 4 h and the generated dark yellow-colored formazan was measured at 440 nm using Synergy HTX Multi-Reader (BioTek, USA). Cell density and morphological changes were photographed under phase - contrast inverted microscope Olympus 1 X 7I (Pittsburgh, PA, USA) at 20X magnification.

### 2.5 Statistical Analysis

Data were analyzed using the Graph Pad Prism 6.2 Software (San Diego, CA, USA). All data points were obtained from the average of at least two independent studies and expressed as mean ± SEM. Inhibitory concentrations (IC_50_s) for lactate efflux and cell viability studies and IG_50_ for growth inhibition studies, were determined by nonlinear regression with lowest 95% confidence interval and R^2^ best fit. The significance of the difference between two groups was determined by unpaired t-test, between control and treated groups using one-way ANOVA followed by Dunnett's multiple comparison's test. Significance of the difference between the control and treated groups is considered at *P = 0.05, ** *P* = 0.01, *** *P* = 0.001, and **** *P* = 0.0001.

## 3. RESULTS

### 3.1 High Throughput Plant Extracts Screening for Lactate Efflux Inhibitors

**Fig. 1:**
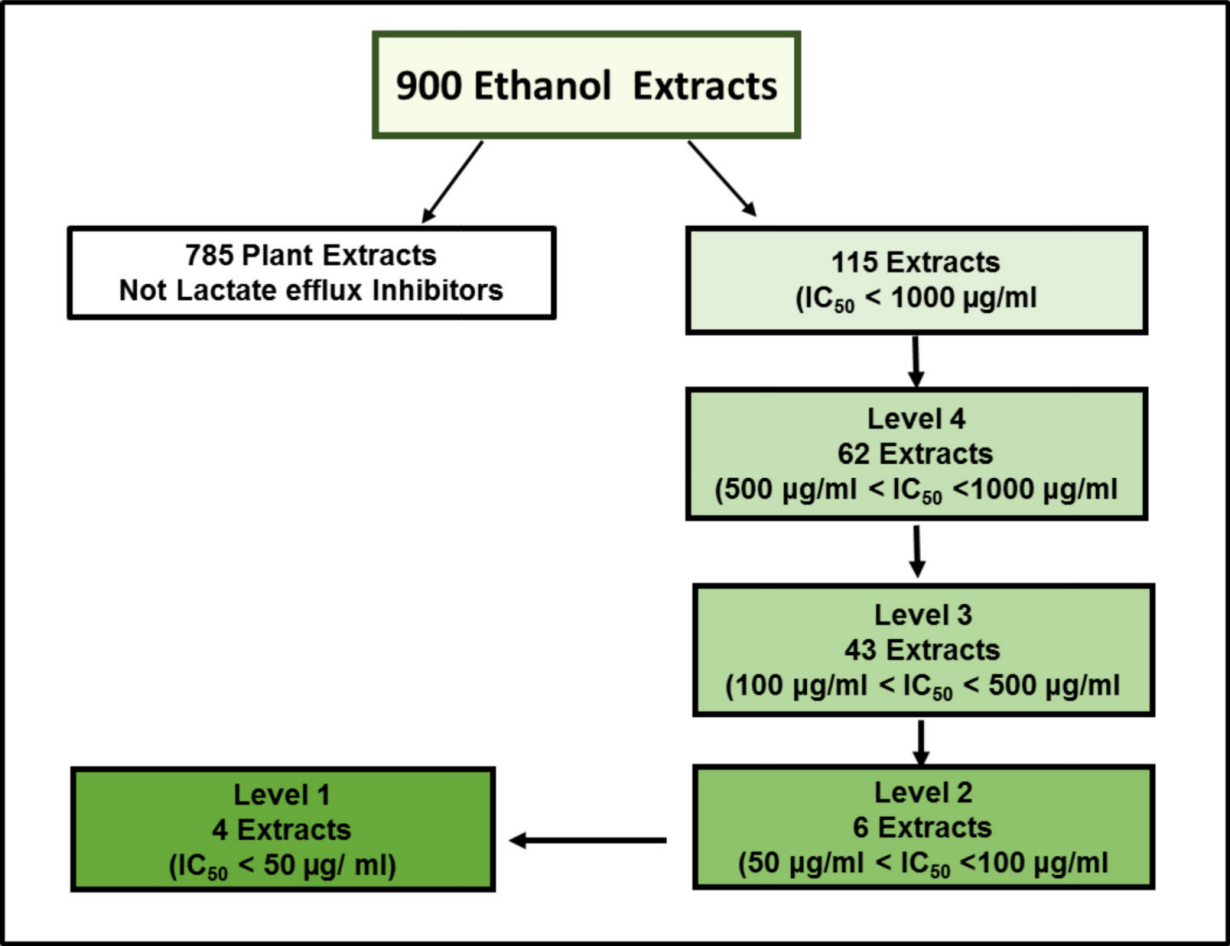
Schematic diagram of high throughput screening for 900-plant ethanol extracts (EE) to identify and rank natural lactate efflux inhibitors in N2-A cancer cells N2-A cellular lactate production of treated cells was compared to untreated normalized average % control total lactate production within 4 h of incubation with each extract. Extracts indicating an IC_50_ <1000 μg/ml were rescreened at lower concentrations (500, 100, and 50 μg/ml). According to the IC_50_s, the potent plant extracts were categorized into 4 levels, and 4 plant extracts were the most potent (IC_50_s < 50 μg/ml) and identified as Bupleurum chinense, Rheum palmatum, Terminalia chebula, and Trillium pendulum.

The high throughput screening of 900 ethanol plant extracts was designed to identify natural potent lactate efflux inhibitors in N2-A cancer cells at four tiers (Plant extract concentration: 50 - 1000 μg/ml). Based on < 50% lactate efflux compare to the control, 785 (87%) of the tested plant extracts were not active and excluded from the study after the first tier. The other extracts (115) were active and categorized according to their potency into four levels (Fig. 1 and Table 1). The fourth level were considered the least potent and included 62 extracts with (500 μg/ml < IC_50_ < 1000 μg/ml). 43 extracts showed average potency (100 μg/ml < IC_50_ < 500 μg/ml) and placed on the third level and 6 extracts showed higher potency (50 μg/ml < IC_50_ < 100 μg/ml) at the second level. Four plant extracts were categorized as the most potent at level 1 (IC_50_ < 50 μg/ml). These plant extracts were identified according to their potency as *Terminalia chebula* (IC_50_ 42.78 μg/ ml), *Bupleurum chinense* (IC_50_ 43.22 μg/ml), *Trillium pendulum* (IC_50_ 49.82 μg/ml), and *Rheum palmatum* (IC_50_ 49.82 μg/ml). Among these four extracts, *Terminalia chebula* was the most potent and therefore, further studies were performed using this plant extract.

### 3.2 TCE Lactate Efflux Inhibition Potency

**Fig. 2:**
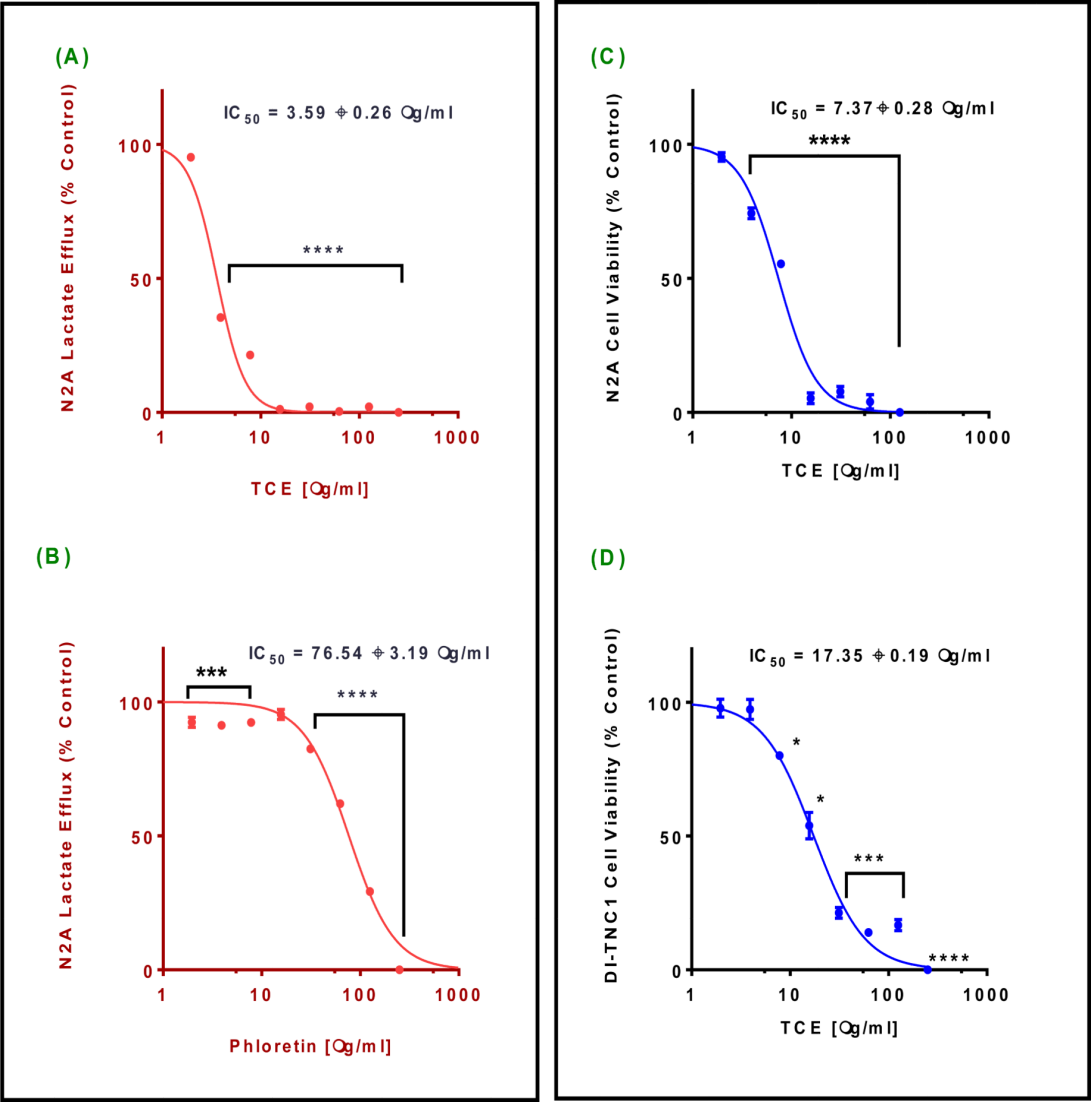
Effect of *Terminalia chebula* (TCE) on lactate efflux and cell viability (A) and (B) are lactate production profile of N2-A cells after 4 h exposure to different concentrations of TCE and phloretin, respectively. (C) and (D) are cytotoxicity profile of N2-A and DI-TNC1 cells after 24 h exposure period to different concentrations of TCE. Statistical analysis of all studies was presented as the mean ± SEM from the average of two independent experiments, n=4 each. IC_50_s are average of two independent studies sigmoidal curves. The significance of the difference between controls vs. treated cells was determined using a one-way ANOVA followed by Dunnett's multiple comparisons test. Significance of difference between control and treatment is considered at *P = 0.05, *** P = 0.001, and **** P = 0.0001

To determine **TCE** potency, we conducted dose-response studies for lactate efflux changes in N2-A cells supernatant. Lactate production was inversely proportional to the increased **TCE** concentrations. Inhibition of lactate efflux was highly significant (*P* = 0.0001), giving IC_50_ value of 3.59 ± 0.26 μg/ml (Fig. 2A). Lactate efflux inhibition was less than 10% in N2-A cells treated with a-cyano-4-hydroxycinammic acid (CHC), at the highest tested concentration (250 μg/ml = 1.32 mM). Meanwhile, phloretin induced highly significant effect (*P*< 0.0001) with IC_50_ 76.54 ± 3.19 μg/ml (279.07 μM). Compare to the calculated IC_50_ of **TCE**, phloretin was less potent by 21.32 fold (Fig. 2B). Similarly, the dose - response of the cytotoxicity studies performed using N2-A cells vs. DI-TNC1 primary cells to assess the safety of **TCE** (Fig. 2 C and D). The data obtained indicated a significant inverse relationship between the viability and the tested concentrations in both cell lines (*P* = 0.0001). Noticeably, **TCE** was 2.35 fold less potent in the primary cells (IC_50_ of 17.35 ± 0.19 μg/ml) compare to N2-A cells (IC_50_ of 7.37 ± 0.28 μg/ml).

### 3.3 TCE Reduces MCTs and CD147 Expression

**Fig. 3:**
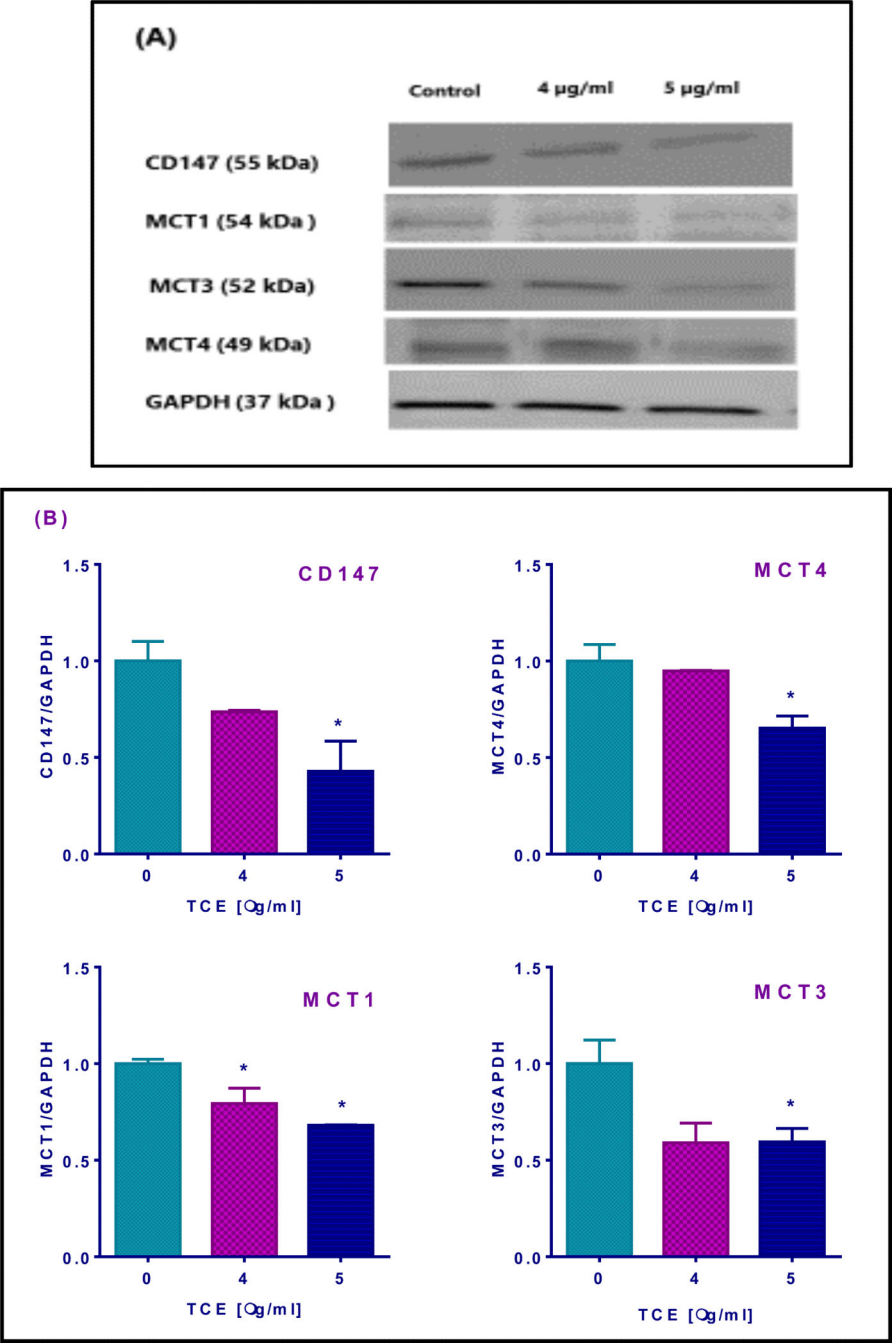
*Terminalia chebula* extract (TCE) effect on the expression of monocarboxylate transporters (MCTs) and their chaperone CD147 in N2-A cancer cells after 4h treatment with concentration range 0 to 5 μg/ml of TCE (A) Indicates the presence of all candidates as detected by their molecular weight compared to the standard protein. The decrease in band intensities appeared precisely at 5 μg/ml, and loading consistency was confirmed by GAPDH. (B) Data obtained from two independent studies showed a significant decrease in protein expression in all candidates at 5 μg/ml. Statistical analysis was presented as the mean SD from the average of two independent experiments. The significance of the difference between the control and treated cell lysates was determined using one-way ANOVA followed by Dunnett's multiple comparisons tests. The significance level was set at *P = 0.05.

To understand the mode of action engaged in lactate efflux inhibition we performed Western blotting for N2-A cell lysates and evaluated protein expressions of monocarboxylate transporters and their chaperone CD147 after 4 h exposure to different concentrations of **TCE**. Antibodies detected the different MCTs, an indication of their presence in N2-A cell line (Fig. 3A). Moreover, at the highest tested dose 5 μg/ml, **TCE-**induced a significant decrease in protein expression (*P* = 0.05), giving 57% reduction in CD147; 35% reduction in MCT4 ; 32% reduction in MCT1; and 41% reduction in MCT3 expression (Fig. 3 B).

### 3.4 TCE Induces Apoptosis, Morphological Changes, and Activates Caspase 3 in N2-A Cells

**Fig. 4:**
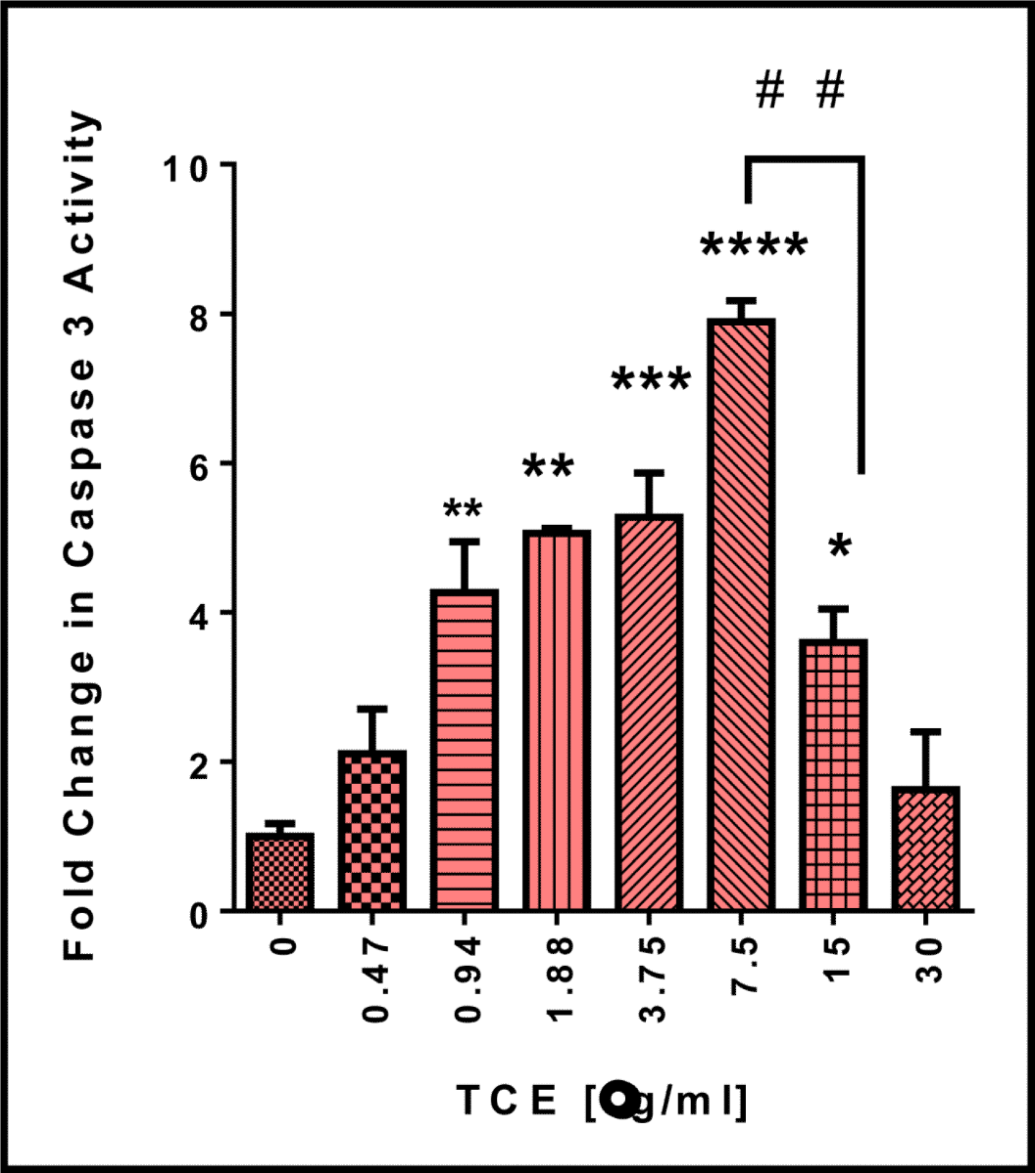
Activation of caspase 3 in N2-A cells by *Terminalia chebula* (TCE) Caspase 3 was measured in the cell lysates of two independent studies with n=3 and expressed as fold increase compares to the control. The significance of the difference between treated cells vs. control. Significance is considered at * P = 0.05, ** P = 0.01, *** P = 0.001, **** P = 0.0001, and ^# #^ P = 0.01.

The change of caspases 3 activity was used as a marker for apoptosis and cell death that might be attributed to lactate efflux inhibition. Cell apoptosis was measured in N2-A cells after 4 h exposure to **TCE.** The results show that a significant increase in caspase 3 activity, in a dose - dependent manner, was detected in the cell lysates (Fig. 4). The significant difference between treated and control cells was detected at 7.5 μg/ml (*P* = 0.0001), giving almost 8 folds’ increase in caspase activity relative to the control cells. Also, a significant decrease was also obtained (^# #^ P = 0.01) at a higher dose (15 μg/ml).

**Fig. 5:**
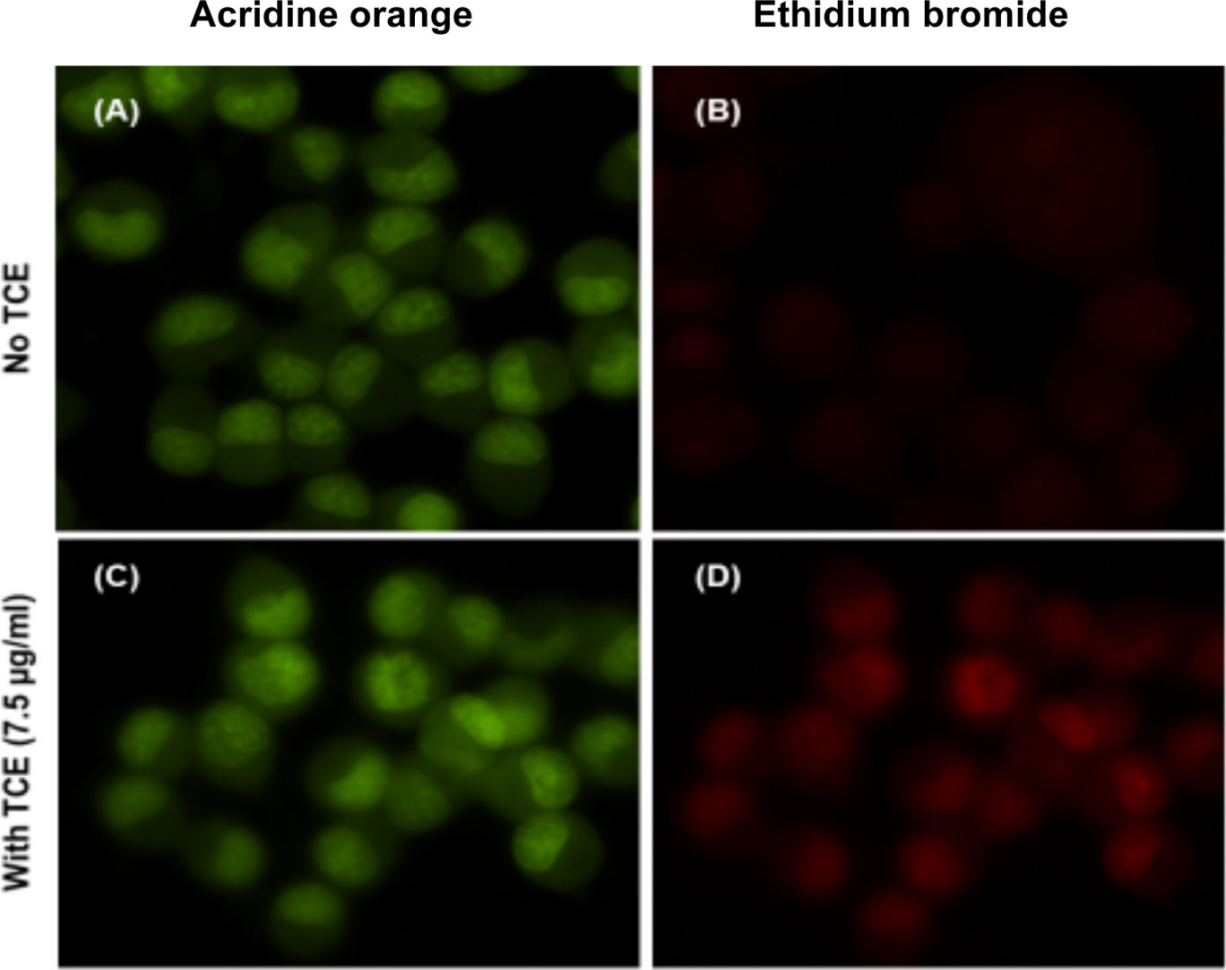
Apoptotic effect of *Terminalia chebula* (TCE) in N2-A cells (A) Control cells stained with acridine orange and appeared with uniform green - stained nuclei. (B) Control cells stained with ethidium bromide. (C) Acridine orange - stained cells treated for 4 h with 7.5 μg/ml of TCE appeared with bright dots at the nuclei as symptoms of early apoptosis. (D) Ethidium bromide stained cells treated for 4 h with 7.5 μg/ml of TCE appeared red color and fragmented and condensed nuclei were detected in late apoptotic cells. Microscopic magnification was 40X.

The apoptosis-related morphological changes of **TCE** were further investigated using acridine orange/ethidium bromide fluorescence assay. Untreated cells appeared with uniformly green nuclei (Fig. 5 A) while different degrees of early and late apoptotic features appeared clearly in cells treated with 7.5 μg/ml (Fig. 5 C and D). Early apoptotic cells appeared with bright green dots in the nuclei, while chromatin condensation and nuclear fragmentation were detected in the late apoptotic stage as cells lose the membrane integrity and incorporate a red color - ethidium bromide.

### 3.5 The Growth Inhibition Effects of TCE

**Fig. 6:**
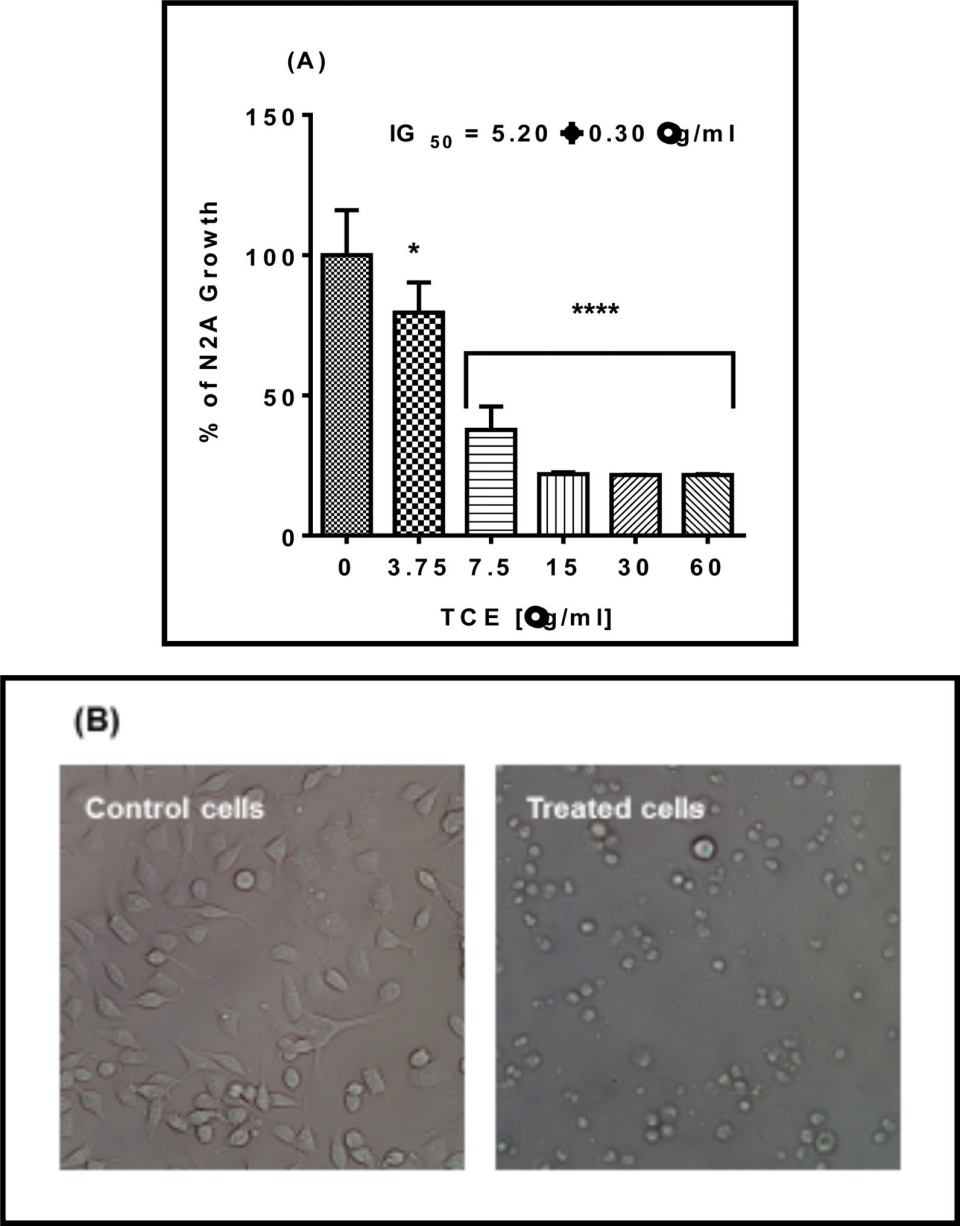
Effect of *Terminalia chebula* (TCE) on N-2A cell growth and morphology (A). Cell growth activity of N2-A treated for 48h with different concentrations of TCE. Statistical analysis is presented as the mean ± SEM of two independent experiments with n=4. The significance of the difference between treated cells vs. control was determined using one-way ANOVA followed by Dunnett's multiple comparisons test. The IG_50_ is the average of two studies sigmoidal curves. Significance is considered at *P = 0.05, and **** P = 0.0001. (B). Phase contrast of N2-A cells treated for 48 h with or without 15.0 μg/ml of TCE and microscope magnification was 20 x objective magnification.

The impact of **TCE** on N2-A cell growth was evaluated at 48 h exposure period. **TCE** decreased cell proliferation in a dose-dependent pattern with a highly significant reduction in cell proliferation (*P* = 0.0001) was observed at the tested concentration of 7.5 μg/ml and above, giving IG_50_ = 5.2 ± 0.30 μg/ml (Fig. 6A). Remarkably, almost 76% reduction in cell proliferation was obtained at 15 μg/ml of **TCE** and remained consistent at the other higher doses. Also, Phase-contrast microscopy revealed that treated cells decreased in numbers and appeared round with shrunk size compared to the control. (Fig. 6B).

## 4. DISCUSSION

Lactate efflux is critical for cancer cell metabolism and proliferation. Thus, targeting lactate produced by cancer cells was the primary goal of this study. Extracts of 900 plants were screened for lactate efflux inhibition in N2-A neuroblastoma cells that are characterized by a high metabolic rate and excess lactate efflux [29]. The extract of *Terminalia chebula* (**TCE**) plant was the most potent extract as lactate efflux inhibitor. The plant, Terminalia chebula Retz, belongs to the family Combretaceae and also called black Myrobalans (English) and Harad (Hindi). The full grown plant is a tall tree up to 80 feet in height, is native to India, known as the ‘King of Medicine’ since it was used in healing many diseases such as heart diseases, asthma, gout, bleeding piles, vomiting, diarrhea, ulcers, sore throat, and dysentery [17]. The extensively studied *Terminalia* species indicate that this plant has a wide spectrum of medicinal effects. The plant was reported to have an antimicrobial [30], antiviral, antimalarial and antifungal [31], antiprotozoal [32], anti-inflammatory, anti-arthritic [33], antidiabetic [34], hepatoprotective [35], antioxidant [36], antianaphylactic [37], antimutagenic [38], and anticancer [39-43] effects. Several studies have also indicated that the methanolic and water extracts of **TCE** have an inhibitory action on the human immunodeficiency virus [44] and immunomodulatory action [45]. Additionally, a recent study using the rat pheochromocytoma (PC12) cell line indicated that the extract of the dried ripe fruit has a neuroprotective effect against ischemia related damage [46]. Several *in vivo* studies on the pharmacological effects of the extract of the Terminalia chebula plant (TCE) were investigated using the rat and the mouse. Many reports indicated the effectiveness of this plant extract as an anti-inflammatory agent [47-48]. Moreover, the chemopreventive effects of TCE in stomach cancer in the rat were reported earlier [49].

Since our primary concern in this study is to evaluate the levels of extracellular lactate as an indication of functional MCTs, we examined the potency of **TCE** comparing to the well- known lactate inhibitors phloretin and CHC [50, 51]. The obtained results indicate that 50% of lactate efflux inhibition in N2-A cell was obtained when cells were treated with 279.07 μM of phloretin. The obtained results are in agreement with the previously reported study that found 300 μM of phloretin inhibited lactate transport in erythrocytes [52]. Interestingly, our data showed a remarkable effect of **TCE** over phloretin. On the contrary, current data did not show a significant inhibitory effect of CHC at the highest tested concentration. In spite of the reported effects of CHC as an MCT1 selective inhibitor [53] by affecting the expression of MCT1 [3], no sufficient information about the impact of CHC on N2-A cells. However, our results agree with previous studies that 5mM of CHC did not inhibit lactate efflux in glial cells [54] and should be at least 10 mM to inhibit MCT efflux in malignant gliomas [55].

Current literature did not report the selective cytotoxicity of **TCE** among different cancer cell lines. However, *Terminalia chebula* was reported as a safe chemopreventive drug within the recommended Ayurvedic specifications [56]. Also, in an *in vivo* study, *Terminalia chebula* dried fruits water extract was found to cause neither acute nor chronic toxicities when tested in male or female rats [57]. These data agree with our cytotoxicity study on DI-TNC1 primary cell line.

To explore the mechanism of action of lactate efflux inhibition by **TCE**, we examined MCT transporters as important pH regulators in high glycolytic solid tumors that mediate lactate transportation across the plasma membranes [58]. Also, the suppression of monocarboxylate transporters is considered the first step in apoptosis [59]. Lactate efflux through MCT4 was previously reported [2]. However, MCT1 and MCT3 might facilitate lactate passing through the plasma membrane under certain conditions [16-17]. On the contrary, MCT2 expression is reduced in highly glycolytic cancer cells [60] since it involves in lactate uptake under normal metabolism [61]. Thus, Western blotting was performed to evaluate the expression of MCT1, MCT3, and MCT4 in treated N2-A cells. Furthermore, the expression of a chaperone to some MCTs was also studied. CD147 is a multifunctional protein and also known as basigin, controlling and regulating energy metabolism of cancer cells [62]. Importantly, it is necessary for MCTs stabilization and expression at the cell membrane [63]. Accordingly, disabling MCTs through disrupting their association with CD147 is considered one of the novel approaches to inhibiting MCTs.

To our knowledge, this is the first study to report on the expression of MCT1, MCT3, and MCT4 and the chaperone CD147 in neuroblastoma N2-A cells. However, previous studies found similar expression of MCT1 in human neuroblastoma cell lines (IMR32, NGP, and SK-N-SH) [29] and MCT4 expression was higher in MDA-MB-231 [64]. Although all proteins under investigation showed a significant decrease in their expression at the highest tested dose of TCE, the highest reduction was observed in CD147 expression. Considering all these findings, we might attribute **TCE** inhibition of lactate efflux to the reduction of CD147 expression more than MCT4 itself. In other words, **TCE** may have inhibited MCT4 function indirectly through CD147 suppression. The role of MCT3 in cancer cells is poorly studied. However, a previous study on the retina of the rat reported MCT3 as lactate efflux transporter [65]. Interestingly, the decrease in MCT1 expression might be another reason for the insignificant lactate efflux inhibitory effect of CHC in N2-A cells, an interpretation that agrees with a previous study since CHC exerts an inhibitory effect on tumors cells expressing MCT1 at the plasma membrane [15].

In the current study, apoptotic effect of **TCE** was confirmed by caspase 3 activity. Caspase 3 is a cysteine protease, and its activation is considered a critical step in cell apoptosis [66]. Our findings are in agreement with earlier studies indicated that quercetin isolated from the fruits of *Terminalia spp* was found to induce apoptotic effects in N2-A cells [67], chebulagic acid was also reported to induce apoptosis in COLO-205 cells [68]. Similarly, apoptosis was reported in human breast cancer MDA-MB-231 treated with pentagalloylglucose and quercetin [69] and HL-60 cells treated with ellagitannins [70]. Current proliferation study was comparable to the previous study that showed a decrease in cell proliferation upon lactate efflux inhibition in breast cancer cells [71]. Despite the differences in the method of extraction, as well as the cell line, the growth inhibition effect was profound by *Terminalia chebula* when tested in various cell lines [39].

## 5. CONCLUSION

Out of 900 ethanol plant extracts screened, *Terminalia chebula* ethanol extract was found to be the most potent lactate efflux inhibitor with the ability to inhibit chaperone CD147 expression and impact the function of monocarboxylate transporters. Furthermore, TCE has growth inhibition and apoptotic effects. The obtained results indicate that the plant *Terminalia chebula* constituent(s) may contain new targets for the management of neuroblastoma. 

## Figures and Tables

**Table 1:** The effect of top ethanol plant extracts as lactate efflux inhibitors in N2-A cells. Cells were exposed 4h to different concentrations of the plant extracts. Compared to lactate production in control cells at the highest dose (1000 μg/ ml), 785-plant extracts were not active. The other plant extracts were categorized according to their potency as following: 62 extracts (500 μg/ml < IC50 < 1000 μg/ml) and ranked as the lease potent, 43 extracts (100 μg/ml < IC50 < 500 μg/ml), 6 extracts (50 μg/ml < IC50 < 100 μg/ml), and 4 ethanol plant extracts (IC50 < 50 μg/ml) and considered as the most potent.

Rank	Common Name	Scientific Name
**Level 1 (IC_50_ < 50 μg/ml)**		
	Beth root	*Trillium pendulum*
	Bupleurum root	*Bupleurum chinense*
	Haritaki fruit	*Terminalia chebula*
	Turkey rhubarb root	*Rheum palmatum*
**Level 2 (50 μg/ml < IC_50_ < 100 μg/ml)**		
	Green tea	*Camellia sinensis*
	Morning glory seeds	*Semen pharbiditis*
	Sancha leaf green tea	*Camellia sinensis*
	Thyme herb	*Thymus vulgaris*
	Witch hazel root	*Hamamelis virginiana*
	Yerba mate leaf	*Ilex paraguarensis*
**Level 3 (100 μg/ml < IC_50_ < 500 μg/ml)**		
	Allspice	*Pimenta dioica*
	Babul chall bark	*Acacia arabica*
	Balm of gilead	*Populus balsamifera L*
	Bay leaf	*Laurus nobilis*
	Bayberry root bark	*Morella cerifera*
	Bhumy amalaki	*Phyllanthus niruri*
	Bilberry leaf	*Vaccinium myrtillus*
	Biota leaves	*Biota orientalis*
	Birch leaf	*Betula alba*
	Bishop's wort	*Stachys officinales*
	Blackberry leaf/root	*Rubus fruticosus*
	Buchu leaf	*Agathosma betulina*
	Buddleia flower bud	*Buddleia officinalis*
	Bushy knotweed rhizome	*Polygonum cuspidatum*
	Butternut bark	*Juglans cinerea*
	Canadian snake root	*Assarum canadense*
	Centaury herb, c/s	*Centaurium erythracea*
	Cleavers herb	*Galium aparine*
	Comfrey leaf	*Symphytum officinale*
	Dogbane leaf	*Apocynum venetum*
	Feverfew leaf and flower	*Tanacetum parthenium*
	Fleeceflower caulis	*Polygonum multiflorum*
	Fossilized teeth	*Dens draconis*
	Fringe bark tree	*Chionanthus virginicus*
	Golden eye-grass rhizome	*Rhizoma curculiginis*
	Gunpowder green tea	*Camellia sinensis*
	Heather flower	*Calluna vulgaris*
	Hyssop flowers	*Hyssopus officinalis*
	Italian spice herbal tea	*Italian spice herbal tea*
	jasmine flavored green tea	*Jasminum officinale*
	Lemon verbena leaf and flower	*Aloysia triphylla*
	Linden leaf	*Tilia europaea*
	Olive leaf	*Olea europaea*
	Osha root	*Ligusticum porteri*
	Paul D'Arko bark	*Tabebuia impetiginosa*
	Pipsissewa leaf	*Chimaphila umbellata*
	Pomegranate husk	*Punica granatum*
	Sassafras root bark	*Sassafras albidum*
	Soap horn thorn	*Gleditsia sinensis*
	*Stone seeds*	*Lithospermum erythrorhizon*
	White sage leaf	*Salvia apiana*
	Wild cherry bark	*Prunus serotina*
	Wild yam root	*Dioscorea villosa*
**Level 4 (500 μg/ml < IC_50_ < 1000 μg/ml)**		
	Acanthopanax root bark	*Acanthopanax gracilistylus*
	Agrimony herb	*Agrimonia eupatoria*
	Akebia fruit	*Fructus akebiae trifoliatae*
	Alkanet root	*Alkanna tinctoria*
	Allspice berry powder	*Pimenta dioica*
	American pennyroyal herb	*Hedeoma pulegioides*
	Anise star seed and flower	*Illicium verum*
	Arjun bark	*Terminalia arjuna*
	Asafoetida, powder	*Ferula assa-foetida*
	Bian u herb	*Polygonum aviculare*
	Black cardamon pods	*Fructus alpiniae oxyphyllae*
	Black henna leaf	*Lawsonia inermis*
	Black pepper fruit	*Piper nigrum*
	Black walnut hull	*Juglans nigra*
	Blood root	*Sanguinaria canadensis*
	Blue verian arial portion	*Verbena hastata*
	Calamus root	*Acorus calamus*
	California poppy arial portion	*Eschscholzia californica*
	Cang Zhu	*Atractylodes chinensis*
	Carpesi fruit mult	*Carpesium abrotanoides*
	Celery seed	*Apium graveolens*
	Chang Shan (Hortensia)	*Dichroa febrifuga*
	Chaparral (greasewood)	*Larrea tridentata*
	Chili peppers flakes	*Capsicum annuum*
	Chinese Clematis Root	*Radix clematidis*
	Chinese thoroughwax	*Bupleurum falcatum*
	Cinnamon twig	*Cinnamomum cassia*
	Corriander seed powder	*Coriandum sativum*
	Cumin seed	*Cuminum cyminum*
	Desert thumb (red thumb)	*Cynomorium songaricum*
	Drgaon's blood	*Dracaena cinnabari*
	Epazote herb (wormseed)	*Dysphania ambrosioides*
	Eucalyptus leaf	*Eucalyptus globulus*
	Evergreen wisteria	*Millettia reticulata*
	Eyebright leaf and stem	*Euphrasia officinalis*
	Figwort herb	*Scrophularia nodosa*
	Fleece flower root	*Polygonum multiflorum*
	Frankincense	*Boswellia resin*
	Gallnut of Chinese sumac	*Melaphis chinensis*
	Galangal root	*Alpinia galanga*
	Gloryvine stem	*Sargentodoxa cuneata*
	Golden root	*Rhodiola rosea*
	Grapeseed extract	*Vitis vinifera*
	Hookweed roots	*Cyathula officinalis root*
	Indian lotus leaf	*Nelumbo nucifera*
	Irish breakfast green tea	*Camellia sinensis*
	Juniper berry, powder	*Juniperus communis*
	Kochia seed	*Kochia scoparia*
	Magnolia flower	*Magnolia denudata*
	Mandrake root	*Podophyllum peltatum*
	Marigold petals	*Calendula officinalis*
	*Notopterygium root*	*Notopterygium incisium*
	Nutmeg powder	*Myristica fragans*
	Orange powder	*Citrus sinensis*
	peppermint leaf	*Mentha piperita*
	Pipsissewa leaf	*Chimaphila umbellata*
	Plantain leaf	*Plantago major*
	Pomegranate Husk	*Punicum granatum*
	Red Henna leaf	*Lawsonia inermis*
	Sancha leaf green tea	*Camellia sinensis*
	Wood-fern, shield fern	*Rhizoma dryopteris*
	Yerba santa leaf	*Eriodictyon californicum*
